# Detection of *Fusobacterium nucleatum* DNA in primary care patient stool samples does not predict progression of colorectal neoplasia

**DOI:** 10.1371/journal.pone.0269541

**Published:** 2022-06-03

**Authors:** Alan Aitchison, John F. Pearson, Rachel V. Purcell, Frank A. Frizelle, Jacqueline I. Keenan

**Affiliations:** 1 Department of Surgery, University of Otago Christchurch, Christchurch, New Zealand; 2 Biostatistics and Computational Biology Unit, University of Otago Christchurch, Christchurch, New Zealand; University of Illinois Urbana-Champaign, UNITED STATES

## Abstract

**Background:**

Carriage of certain bacterial species may represent potential biomarkers of colorectal cancer (CRC). Prominent among these is *Fusobacterium nucleatum*. We explored the association of *F*. *nucleatum* DNA in stool samples with the presence of colonic neoplastic lesions in a cohort of primary care patients, and compared our findings with those from an unrelated cohort of colonoscopy patients followed clinically over time.

**Methods:**

Carriage rates of *F*. *nucleatum* in stool samples were assessed in 185 patients referred for a faecal immunochemical test (FIT) by their general practitioners (GPs). Comparisons were made with stool samples from 57 patients diagnosed with CRC and 57 age-matched healthy controls, and with tissue samples taken at colonoscopy from 150 patients with a decade of subsequent clinical follow-up.

**Findings:**

*F*. *nucleatum* DNA was found at a high rate (47.0%) in stool samples from primary care patients, and more often in stool samples from CRC patients (47.4%) than in healthy controls (7.0%), (P = 7.66E-7). No association was found between carriage of *F*. *nucleatum* and FIT positivity (P = 0.588). While evidence of stool-associated *F*. *nucleatum* DNA was significantly more likely to indicate a lesion in those primary care patients progressed to colonoscopy (P = 0.023), this finding did not extend to the progression of neoplastic lesions in the 150 patients with a decade of follow up.

**Conclusion:**

The finding of *F*. *nucleatum* DNA at similar rates in stool samples from patients diagnosed with CRC and in primary care patients with pre-cancerous lesions supports growing awareness that the presence of these bacteria may be a biomarker for increased risk of disease. However, molecular evidence of *F*. *nucleatum* did not predict progression of colonic lesions, which may lessen the utility of this bacterium as a biomarker for increased risk of disease.

## Introduction

Colorectal cancer (CRC) is a considerable health burden globally, being the second most diagnosed cancer in women and third in men [1]. CRC is usually surgically curable in the early stages (I and II) of the disease, with a 5-year relative survival of about 90% [2]. The key to a good prognosis is early diagnosis. This remains an issue however, not least because patients with early stage disease have no symptoms or present with non-specific symptoms in primary care that may require multiple visits before referral for investigation. Accordingly, development of accurate screening tools could considerably reduce the burden of CRC.

The adenoma-carcinoma sequence is well described and, as such, the adenoma provides a target for screening for precancerous lesions. Colonoscopy still provides the most reliable method for screening for adenomas however this relatively expensive, resource-intensive and invasive procedure and as such is not a good population screening tool. Accordingly, biomarkers of early-stage disease that identify at-risk individuals, who would benefit from clinical investigation, are needed. The most widely used non-invasive screening test is the faecal immunochemical test (FIT), which is based on the detection of faecal haemoglobin (f-Hb) in stool samples. While this test has utility as a population-based screening tool, it has limited ability to detect small adenomas (< 1 cm in size) in a primary care setting [3]. While stool-based DNA testing may be more sensitive than FIT with regard to detection of larger lesions, it also lacks the sensitivity to detect small adenomas [4]. Accordingly, other biomarkers of early-stage disease are needed.

There is growing evidence of an association of gut bacteria with CRC. Globally, changes in the composition of gut microbiota (dysbiosis) have been described [5, 6], while an increasing number of studies find that certain species of gut microbiota carrying a range of virulence factors are more prevalent in individuals with CRC when compared to age-matched healthy controls. These bacterial species include enterotoxigenic strains of *Bacteroides fragilis* (ETBF) [7], and strains of *E*. *coli* that carry the *pks* gene cluster encoding the synthesis of the colibactin genotoxin [8]. Long-term colonic carriage of ETBF is associated with significant risk of developing low-grade colonic dysplastic lesions [7] and colonic carriage of *pks*+ *E*. *coli* is reportedly increased in colon cancer [9]. Moreover, animal modelling has been used to illustrate how the *B*. *fragilis* toxin and *E*. *coli* colibactin toxin may work together to promote colon cancer [10]. *Fusobacterium nucleatum*, a common member of the oral microflora, has also been associated with CRC [11–13]. While it has been argued that *F*. *nucleatum* may be an opportunistic pathogen in CRC [14], other evidence supports active involvement of these bacteria in colonic oncogenesis by recruitment of tumour-infiltrating myeloid cells [15] and/or by activation of the Wnt signalling pathway via interaction of *F*. *nucleatum* FadA protein with E-cadherin [16].

Here we investigate the prevalence of *F*. *nucleatum* carriage in a cohort of 185 patients presenting in primary care with bowel symptoms, and correlate the presence of this potential driver of colorectal carcinogenesis with the presence of f-Hb in the same stool samples [3] and, where possible, clinical follow up.

## Methods

**Fig 1:**
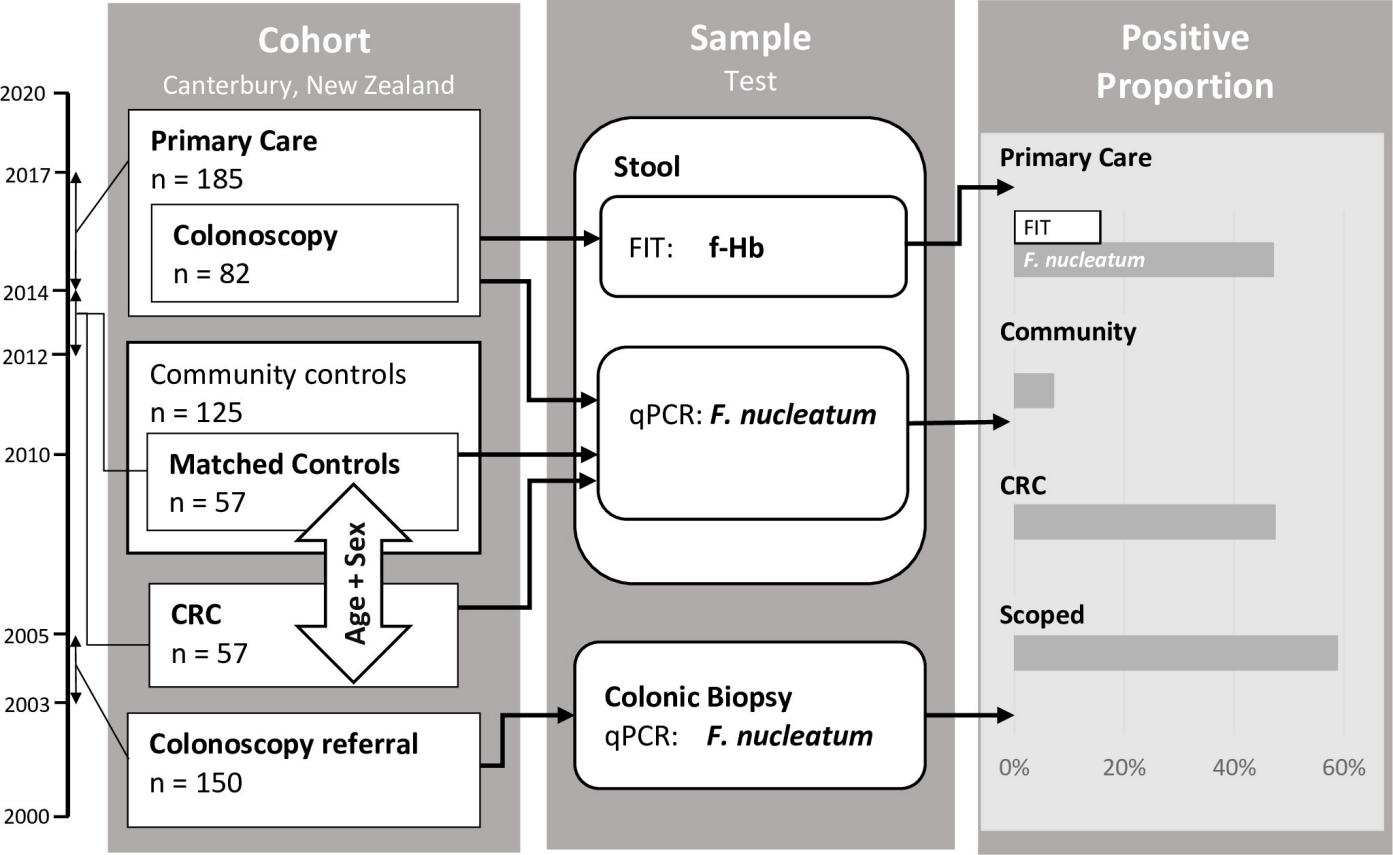
Schematic of study design. Left panel, the four cohorts used in the study with approximate years of collection indicated on the far left; centre panel, analyses performed on cohorts; right panel, graphic representation of results of analyses for each cohort.

A total of four cohorts were investigated in this study, as detailed below. These included stool samples from patients presenting in primary care, unrelated stool samples from age-matched CRC patients and self-reporting healthy community controls, and mucosal biopsies from unrelated patients referred for colonoscopy (Fig 1).

### Primary care patient cohort

One hundred and eight-five patients presenting to their general practitioners (between 2014 and 2017) with bowel problems (hereafter referred to as primary-care patients) and subsequently referred for FIT (to detect the presence of f-Hb) gave written informed consent for their stool samples to also be screened for bacterial biomarkers, and for possible clinical follow-up. This study was approved by the University of Otago Human Ethics Committee (H14/019). Samples were stored at -80°C until DNA extraction.

### Unrelated stool sample cohorts

For comparison, stool samples from two age-matched cohorts were also investigated for molecular evidence of *F*. *nucleatum*. These included samples from 57 individuals, diagnosed with CRC between 2012 and 2014, using standard endoscopic, histological or radiological criteria, who provided a stool sample prior to surgery. Patients found to have had pre-operative chemo-radiation therapy were not included in the study. Additionally, 57 stool samples from a cohort of 125 collected from healthy volunteers who self-reported no evidence of bowel problems at the time of sampling (healthy controls) were age-matched to the CRC patients. All gave written informed consent for their stool samples to be screened for bacterial biomarkers. The CRC patient and healthy control cohorts were initially collected to determine carriage rates of ETBF in the cohorts [17] (Southern Health and Disability Ethics Committee (URA/12/02/005/AM03). The three stool sample cohorts (primary care, CRC and healthy controls) are described in S1 Table in S2 File. Stool samples were stored at -80°C until DNA extraction.

### Colonic biopsy cohort

For further comparison, mucosal biopsies collected from 150 patients referred for colonoscopy at our institution between February 2003 and August 2005 were also investigated for molecular evidence of *F*. *nucleatum*. Patients provided written informed consent for tissue to be collected from up to four different sites in the colon: A, terminal ileum; B, caecum; C, transverse colon; D, recto-sigmoid colon, and for monitoring of clinical follow-up over time (Upper South A Regional Ethics Committee CTY/02/08/132). Details of this cohort are described in S2 Table in S2 File. The samples taken for analysis were macroscopically normal, i.e. no overtly dysplastic, polypoid or cancerous tissue samples were used. Patients had not had previous colonic resections. The samples were frozen in liquid nitrogen and transferred to -80°C until DNA extraction.

Follow-up data was available for 134 patients up to June 2015 (10 to 12 years). Sixteen patients were lost to follow-up during this period, including four who died of CRC, 11 who died of other causes, and one patient who moved to a different country. Clinical data available for the remaining patients at the time of sampling and during this follow-up period included development of CRC, number and type of polyps, presence and type of dysplasia, and side (left or right) of colonic disease, diagnosed from this or subsequent colonoscopies; 75 patients had one or more subsequent colonoscopy.

### Sample preparation

DNA was extracted directly from 100 mg aliquots of individual stool samples using a commercially available kit (Dynabeads DNA DIRECT™ Universal extraction kit, Life Technologie AS, Oslo, Norway). This extract was diluted 100-fold to reduce any inhibitory factors. A different kit (High-Pure PCR Template Preparation Kit, Roche, Nonnenwald, Germany) was used, as per the manufacturer’s instructions, to extract DNA from the biopsies. Purified DNA was quantified using the NanoDrop 2000c spectrophotometer (Thermo Scientific, Asheville, NC, USA), with 260/280 absorbance ratios being between 1.7 and 2.1 for all extracted samples. All extracts were stored at -20°C.

### Stool sample PCR

SYBR-Green chemistry was used to detect evidence of *F*. *nucleatum* in the stool samples, as described previously [17]. Briefly, 25–35 ng of DNA was used along with 0.5 μM of each primer, 5 μl PerfeCTa SYBR Green Fastmix (Quantabio, Beverly, MA, USA) and 1.5 μl H_2_O in a 10μl reaction run on a LightCycler 480 thermocycler (Roche Diagnostics, Indianapolis, IN, USA) for one 5 min cycle of 95°C followed by 45 cycles of 95°C for 10 sec, 57°C for 10 sec and 70°C for 15 sec. Primer details for the *F*. *nucleatum nusG* are shown in S3 Table in S2 File. *Fusobacterium nucleatum subsp*. *nucleatum* (ATCC 25586) was used as a reference strain and a standard curve was made using extracted DNA from this reference strain according to the method of Dolezel *et al* [18]. *F*. *nucleatum* has a genome size of 2.4Mb and a single *F*. *nucleatum* genome weighs 2.43 fg (2.4 Mb/987 Mb [1pg of double-stranded DNA] = 0.00243 pg) Therefore, 1 ng of *F*. *nucleatum* DNA contains approximately 411,523 copies of the genome (1000pg/0.00243 pg). Samples were considered positive if two of the three replicates amplified *F*. *nucleatum* DNA.

### Incidence of *F*. *nucleatum* in colonic biopsies analysed by qPCR

TaqMan probes (S4 Table in S2 File) designed to detect *F*. *nucleatum nusG* gene and a reference gene, *PGT* [12], were used to screen genomic DNA isolated from the colonic biopsies. Each reaction consisted of 25–35 ng of genomic DNA, 5 μl of TaqMan Fast Advanced Master Mix (Applied Biosystems), and 0.5 μl TaqMan primer/probe (Thermo Fisher) in a 10 μl reaction. A LightCycler480 thermocycler was used, and thermal cycling conditions were as follows: 1 cycle of 95°C for 10 mins, followed by 50 cycles of 95°C for 10 secs and 60°C for 30 secs. All reactions were performed in triplicate. DNA extracted from *F*. *nucleatum* subsp. *nucleatum* (ATCC 25586) was used as a positive control.

### Faecal Immunochemical Test (FIT)

A qualitative (one-step membrane cassette) immunoassay was used for detecting f-Hb in each stool sample (Ngaio Diagnostics Ltd, Nelson, New Zealand). This assay detects human haemoglobin above 50 μg of f-Hb per gm of faeces, and is shown to be specific for human haemoglobin.

### Statistical analysis

For stool samples, counts of positive samples were compared between 3 cohorts, healthy controls, primary care and CRC patients using Fisher exact tests. Counts of positive samples between control and both patient cohorts (CRC and primary care) were compared with odds ratios and 95% Wald confidence intervals using Fisher exact P-values. For comparisons with zero counts, the Haldane Anscombe correction was applied prior to calculating odds ratios and confidence intervals. The association of biopsy positivity with location and outcome was assessed using generalized mixed effects logistic regression including fixed effects for age and sex and a random effect for subject, for details see Purcell *et al*. [7]. Abundance was compared by by Welch’s one-way test for differences in means assuming unequal variances on log transformed values followed by posthoc t-tests with P values corrected for multiple comparisons by the Bonferroni method. All tests were 2-sided and considered statistically significant at P<0.05. Analysis was performed in R 4.0.5, using the epiR package.

## Results

### Stool sample analysis

**Fig 2:**
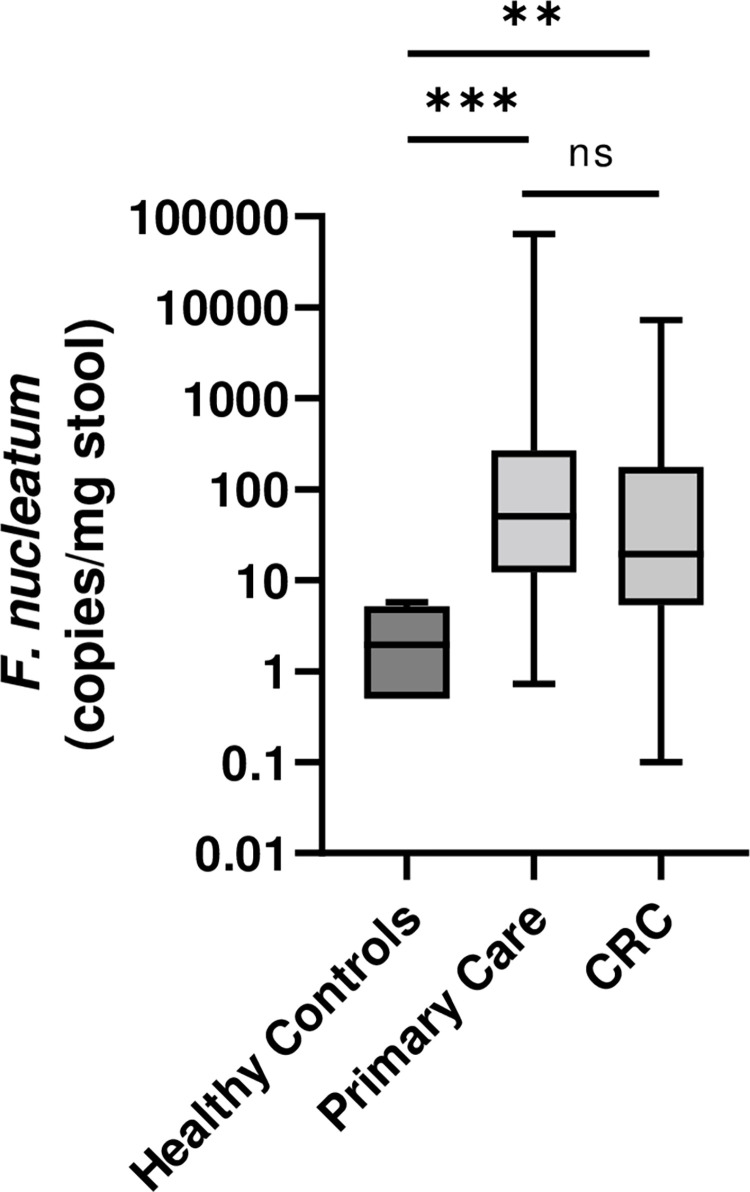
Abundance of *F*. *nucleatum* in stool samples. *F*. *nucleatum* abundance is significantly higher in primary care and CRC stool samples compared to healthy controls (P = 0.0008 and P = 0.0023, respectively) but is not significant between primary care and CRC cohorts, P = 0.341. Values for individual samples are shown in S1 File.

Across cohorts, *F*. *nucleatum* DNA was detected more often in the stools of patients than in healthy controls (Fig 2, Table 1). PCR detected evidence of *F*. *nucleatum* DNA in 87/185 of the primary care-patient samples and 27/57 cancer patient samples, significantly greater than in 4/57 of the age-matched healthy controls (P = 3.02x10^-9^, 7.66x10^-7^ respectively).

**Table 1:** *F*. *nucleatum* positivity relative to a healthy control cohort.

	Cohort	Positive	(%)	*OR*	*[95% CI]*	*P*
**Controls**	57	4	(7.0)			
**GP Patients**	185	87	(47.0)	11.76	[4.09,33.83]	**3.02E-9**
**CRC Patients**	57	27	(47.4)	11.93	[3.81,37.35]	**7.66E-7**

Count of positive samples in patient cohorts compared to controls by Odds Ratios (OR) with 95% Confidence Intervals (CI) and P values.

There was evidence for differences in the abundance of *F*. *nucleatum* across the 3 cohorts (F(2,12.6) = 25.7, P = 0.00004), with both primary care and CRC cohorts showing significantly more abundant *F*. *nucleatum* than healthy controls, P = 0.0008 and 0.0023 respectively, while the difference detected between primary care and CRC cohorts was not statistically significant, P = 0.341, where P values have been corrected for multiple comparisons.

### Clinical investigation

Within the primary care cohort, 82 of the 185 patients subsequently progressed to further investigation that included colonoscopy. Thirty-two of these patients (39%) were found to have evidence of lesions that included CRC (n = 2) and polyps (n = 33) (Table 2). Seven patients presented with lesions greater than 1 cm in size, including the two patients identified with CRC, four patients with tubular adenomas (all reported as having low-grade dysplasia), and one patient found to have multiple sessile serrated adenomas. The other 25 patients had lesions of less than 1 cm while the remaining 50 patients were reported as having a normal colonoscopy. Patients with diverticulosis were considered normal if no evidence of lesions was found.

**Table 2:** Association of stool-associated *F*. *nucleatum* DNA with clinical lesions in the primary care cohort progressed for clinical investigation.

Diagnosis	Any lesion at colonoscopy (n = 32)	*F*. *nucleatum* positive (n = 21)	*F*. *nucleatum* negative (n = 11)
CRC	2	1	1
SP	14	11	3
TA	15	12	3
TVA	4	1	3

CRC, colorectal cancer; SP, serrated polyp; TA, tubular adenoma; TVA, tubulovillous adenoma

Twenty-one of the 40 patients in primary care with evidence of *F*. *nucleatum* DNA in their stool samples and who progressed to colonoscopy were found to have evidence of lesions. The majority of these patients (n = 19) presented with polyps that were less 1 cm in size. Polyps included tubular adenomas, tubulovillous adenomas and serrated polyps (including hyperplastic polyps and sessile serrated adenomas). These were reported in 12,1 and 11 patients, respectively, reflecting histological evidence of more than one polyp subtype in several patients. Ten of these patients were found to have polyps with evidence of low-grade dysplasia. The two patients with lesions greater than 1 cm were found to have multiple sessile serrated adenomas and CRC, respectively. Eleven patients with no evidence of *F*. *nucleatum* DNA in their stool samples were also progressed to colonoscopy (Table 2). While the types of clinical lesions found in the two groups were similar, the presence of *F*. *nucleatum* DNA in the stool was associated with a significantly greater risk of having any lesion found (OR = 3.11, 95% CI [1.23,7.87], P = 0.023).

We have previously reported FIT positivity (>50 μg of Hb per gm of faeces) in 29 of the 185 samples collected from patients in the primary care cohort [3]. Across this cohort, 15 of the 29 patients who had evidence of faecal haemoglobin in their stool sample also had evidence of *F*. *nucleatum* DNA (52%). There was no association of *F*. *nucleatum* positivity with regard to f-Hb status (P = 0.588).

To further investigate the proposed link between colonic carriage of *F*. *nucleatum* and lesions in the colon we looked retrospectively for molecular evidence of *F*. *nucleatum* in biopsies collected from up to four colonic sites from 150 patients undergoing colonoscopy. The age of these patients at the time of the procedure ranged from 19–88 years (mean = 55 years) and there were 100 females and 50 males (S2 Table in S2 File). Previous medical history, along with follow-up medical reports and subsequent colonoscopies were used to generate clinical characteristics for the cohort. Eleven patients had previously diagnosed CRC with an additional nine diagnosed at the time of colonoscopy or during the follow-up period, giving a total of 15% of patients with CRC. Sixty-six patients were diagnosed with having polyps, 23 of these reported as having more than one type of polyp present. As above, the types of polyps described were tubular adenomas, tubulovillous adenomas and serrated polyps, and were reported in 35, 16 and 40 patients, respectively. Low-grade dysplasia was reported in 19/150 patients and high-grade dysplasia in 9/150. A total of 77 patients were reported to have at least one colonic neoplastic lesion (dysplasia, polyps, adenomas, or CRC), and this was reported to be right sided (ascending) in 19/77, left sided (descending) in 45/77, and in both sides in 13 patients. The remaining 73 patients were not diagnosed with any of the lesions being investigated in this study (S2 Table in S2 File).

### Concordance of *F*. *nucleatum* in colonoscopy mucosal biopsies

The presence of *F*. *nucleatum* was confirmed in colonoscopy samples from 88/150 patients (58.7%) following qPCR on DNA samples from up to four colonic sites. In 56 of these 88 patients each sampling site was positive for *F*. *nucleatum* DNA. The remaining 32 patients had at least one colonic site at which *F*. *nucleatum* DNA was undetectable. Sixty-two patients were negative for *F*. *nucleatum* DNA at all sites tested. Hence 118 out of 150 of patients had samples that were either *F*. *nucleatum* positive or negative at all sites, a raw concordance of 79%.

### Association of *F*. *nucleatum* with clinicopathological characteristics

Univariate logistic regression analysis was used to determine associations between *F*. *nucleatum* positivity and clinicopathological parameters of the colonic biopsy cohort (Table 3). No significant associations were seen between *F*. *nucleatum* positivity and the presence of CRC, serrated polyps, high-grade dysplasia, low grade dysplasia, tubular adenomas or tubulovillous adenoma (*P*-values *>* 0.05). Analysis of the association of *F*. *nucleatum* positivity showed that the presence of *F*. *nucleatum* did not differ significantly by colonic site (Table 3). For comparison, we previously reported ETBF to be significantly more likely to be present in more distal samples (Table 3). Recto-sigmoid samples were more likely to be positive than transverse colon in ETBF-colonised individuals, and transverse colon more likely to be positive than caecal samples (P = 0.001). There was no evidence that *F*. *nucleatum* was associated with either presence or location of colonic disease. Likewise, no association was found between *F*. *nucleatum* positivity and patient age (P = 0.19) or gender (P = 0.90).

**Table 3:** Association of *F*. *nucleatum* and ETBF positivity with clinical lesions in the colonic biopsy cohort, adjusted for age and gender.

		*F*. *nucleatum* (this study)			ETBF^7^		
		OR	95% CI	P value	OR	95% CI	P value
**Location**							** **
Site	Recto-sigmoid	1		0.298	1		**0.0010** *
	transverse	1.86	[0.84,4.12]		0.69	[0.21,2.26]	
	caecum	1.47	[0.65,3.30]		0.09	[0.02,0.41]	
**Diagnosis**							
CRC		0.766	[0.88,8.07]	0.087	0.84	[0.30,2.33]	0.738
SP		1.74	[0.82,3.85]	0.152	2.79	[1.31,6.16]	**0.007** *
LGD		1.02	[0.38,2.82]	0.958	4.51	[1.53,16.58]	**0.005** *
HGD		0.97	[0.24,4.17]	0.966	1.98	[0.49,9.77]	0.347
TA		1.13	[0.52,2.51]	0.766	2.43	[1.11,5.58]	**0.027**
TVA		0.53	[0.18,1.53]	0.241	1.76	[0.61,5.47]	0.294

CRC, colorectal cancer; SP, serrated polyp; LGD, low-grade dysplasia; HGD, high-grade dysplasia; TA, tubular adenoma; TVA, tubulovillous adenoma; ETBF, enterotoxigenic *Bacteroides fragilis*.

* significant after adjustment for multiple comparisons.

## Discussion

This study found that the proportion of primary care patients with evidence of *F*. *nucleatum* DNA in their stool samples was similar to that found in CRC patients, and significantly higher than the carriage rate detected in the self-reporting healthy controls. While a number of studies have to date also reported an increased presence of *F*. *nucleatum* DNA in stool samples from patients presenting with adenomas and CRCs [12, 15, 19], this is the first study to extend this finding to patients presenting in primary care with bowel symptoms. We found molecular evidence of *F*. *nucleatum* in approximately 50% of stool samples from these individuals. This is in marked contrast to samples from age-matched self-reporting healthy individuals, where only 7% were found to be positive. Collectively, these findings reinforce the growing awareness that screening for *F*. *nucleatum* DNA may offer the opportunity for early identification of patients who would potentially benefit from clinical investigation and/or ongoing surveillance [20].

Bacteria in the mucosa of the colon are not necessarily well represented in stool, as exemplified by reportedly higher rates of ETBF carriage detected in the colonic mucosa in CRC patients [21] than in patient stool samples [17, 22]. Our finding however of a highly significant difference in the carriage rate of *F*. *nucleatum* DNA in CRC patient stool samples when compared to stool samples from healthy controls confirms other reports that suggest these bacteria are well represented in the lumen of the gut [12, 15, 19], in addition to being present in tissue samples as shown here and by others [11, 12, 15, 23]. Moreover, the observation that the relative abundance of *F*. *nucleatum* DNA in the in the primary care patients was notably higher than that detected in the stool samples from CRC patients reinforces the idea that ongoing surveillance of patients with molecular evidence of *F*. *nucleatum* in stool samples may be warranted.

Current thinking is that Fusobacteria may contribute to tumorigenesis via an inflammatory-mediated mechanism [15], a theory enhanced by the finding that Fusobacterium strains taken from inflamed tissue of IBD patients exhibited increased invasive potential compared to strains found in non-inflamed tissue [24]. More recently, studies suggest that *F*. *nucleatum* may promote CRC development by suppressing aspects of cell-mediated host immunity, and that the notable association between high-level colonisation by these bacteria and MSI-H tumours [23, 25] may relate to a reported association between the abundance of *F*. *nucleatum* in mucosal biopsies and a finding of serrated polyps at colonoscopy [26]. These studies do not, however, confirm a role for these bacteria in initiating as opposed to driving the serrated polyp pathway (or indeed colorectal carcinogenesis). Thus, while patients presenting in primary care with bowel symptoms were found to be significantly more likely to have molecular evidence of *F*. *nucleatum* in their stool samples when compared to healthy controls, these findings do not address cause or effect [27].

The premise that molecular screening for faecal microbiota such as *F*. *nucleatum* may complement FIT in identifying at risk individuals and/or at-risk populations who would benefit from clinical investigation has been explored [19, 28, 29]. Our study failed to find a significant association between molecular evidence of *F*. *nucleatum* and detection of f-Hb in the same stool samples. This is in contrast to the study by Wong *et al*. that showed quantitation of faecal *F*. *nucleatum* improved the diagnostic performance of the FIT with regard to detecting advanced adenomas [19]. This may, in part, reflect the cut off value of the FIT assay, which was notably lower in the Wong study than the threshold of the assay used here (20 μg and 50 μg of Hb per gm of stool, respectively). A recent study reports a negative FIT at a threshold of 2 μg Hb/gm can effectively rule out CRC [30] while the NICE (National Institute for Health and Care Excellence) recommend a threshold of 10–15 μg Hb/gm for triaging patients presenting in primary care with bowel symptoms [31]. However, Grobbee *et al*. [29] also failed to find any association of *F*. *nucleatum* in FIT positive samples from patients with colonic lesions, despite using a FIT with a cut off of 10 μg Hb per gram of stool. It is also possible however that these findings may reflect the reported association between *F*. *nucleatum* and the colorectal serrated pathway [26], coupled with the finding that FIT has poor sensitivity for sessile serrated polyps [32, 33]. To explore this further we examined the histology reports from the 82 of the 185 primary care patients subsequently referred for colonoscopy. Of the 32 patients found to have lesions, most were small polyps (<1 cm) and there was no association of molecular evidence of *F*. *nucleatum* with serrated lesions compared to other neoplasia or a normal colon. While the small numbers in our study preclude more meaningful analysis, the findings are similar to those reported by Grobbee *et al*. [29] who reviewed the findings of 200 FIT positive samples from patients and also failed to find any association between *F*. *nucleatum* and clinicopathological findings at colonoscopy.

We did however find that molecular evidence of *F*. *nucleatum* in stool was associated with a significantly higher risk of having any lesion found. To investigate this further, we looked for the presence of *F*. *nucleatum* DNA in an unrelated cohort of colonic biopsies collected from 150 patients between 2003 and 2005 originally to look for evidence of *Helicobacter* spp. [34] and more recently screened for the presence of ETBF [7]. Clinical data was available for 134 of these patients, both at the time of sampling and during the 10–12 year follow-up period, and included development of CRC, the number and type of polyps, and the presence and type of dysplasia [7]. We found no significant association between clinicopathological features and colonisation with *F*. *nucleatum*. Interestingly, Zakular *et al*. [35] report a similar finding using a 16S rRNA gene sequencing approach. Specifically, the presence of *F*. *nucleatum* was not significantly associated with the development of serrated polyps in our study. This finding was unexpected, given recent studies that find colonic carriage of *F*. *nucleatu*m is associated with MSI-H [23, 36] or CIMP+ [26, 36] subtypes of CRC that are considered to develop through the serrated neoplasia pathway [37]. There was also no evidence that the presence of *F*. *nucleatum* was more likely to be detected in the proximal as opposed to the distal bowel, as reported elsewhere [26, 38]. It is possible that these differences may, in part reflect our reporting of the presence as opposed to the relative abundance of *F*. *nucleatum* in these samples and we acknowledge this as a potential limitation of our findings.

Levels of the *F*. *nucleatum nusG* gene and a reference control, prostaglandin transporter (PGT), were simultaneously measured in this study using a probe that detects *F*. *nucleatum* subsp. *nucleatum* [7, 11]. It was noted however that the *F*. *nucleatum nusG* primers used in the probe have differing sequence similarities to each of the four *F*. *nucleatum* subspecies, only two of which (nucleatum and animalis) are considered disease-associated [39]. While the forward primer had 100% identity across the entire length of the primer with all four subspecies, the reverse primer did so only for the *F*. *nucleatum* subsp. *animalis* (strain ChDC F332). Each of the remaining subspecies, *F*. *nucleatum* subsp. *nucleatum* (strain ATCC 25586), *F*. *nucleatum* subsp. *polymorphum* (strain ChDC F306) and *F*. *nucleatum* subsp. *vincentii* (strain KCOM 2931) contained three mismatches within the 5’ half of the reverse primer. Accordingly, while minor enough not to affect detection of *F*. *nucleatum* per se, it is likely that the use of this reverse primer may sufficiently affect the dynamics of the PCR to preclude assessment of relative abundance between samples. Whereas one study to date reports *F*. *nucleatum* subsp. *animalis* is the predominant *F*. *nucleatum* subspecies in CRC specimens [40], this is an area that warrants further investigation.

A potential limitation of our study might be seen as our focus on a single bacterial candidate as a novel biomarker for non-invasive diagnosis of CRC [41]. Our goal, however, in screening for a potential bacterial biomarker (alone, and in combination with FIT) was to identify symptomatic patients presenting in primary care who should be progressed for clinical investigation [42]. Accordingly, our primary care cohort did not include asymptomatic individuals [43]. Other potential limitations of this study include statistical power which was adequate for the primary comparison but limited in sub-group analysis by the low incidence of *F*. *nucleatum* in control samples and biases in presentation and referral criteria, for example by ethnicity which is better described elsewhere [44]. Additionally, the use of samples collected across different calendar times and from different cohorts with different selection criteria might also be considered a limitation of our study, although the findings suggest otherwise.

In summary, in addition to cancer and healthy control cohorts, this study uniquely investigates the carriage of *F*. *nucleatum* in a primary care cohort referred for FIT. Our results suggest that while *F*. *nucleatum* is detected at higher rates in the stool samples of individuals presenting in primary care with bowel problems than in healthy individuals, it may not necessarily by itself be a biomarker of lesions that have the potential to drive colon carcinogenesis.

## Supporting information

S1 File Abundance of *F*. *nucleatum* in patient cohorts. (XLSX)

S2 File (DOCX)
