# Supplementary material for: Detection of Fusobacterium nucleatum DNA in primary care patient stool samples does not predict progression of colorectal neoplasia
Source: PLoS One. 2022 Jun 3;17(6):e0269541. doi: 10.1371/journal.pone.0269541 (PMC9165787; doi:10.1371/journal.pone.0269541)
Supplement: S2 File — (DOCX) [file pone.0269541.s002.docx]

**Supplementary Data**

**S1Table.** **Numbers and average age of cohorts who provided faecal samples**

| **Cohort** | **Number** | | **Gender**  **(M:F)** | **Median age**  **(interquartile range)** |
| --- | --- | --- | --- | --- |
| Healthy controls | | 57 | 25:32 | 64 (40-74) |
| GP patients | | 185 | 91:94 | 59 (51-70) |
| CRC patients | | 57 | 33:24 | 72 (63-76) |

**S2 Table.** **Patient and clinical characteristics of the colonoscopy cohort [1]**

|  |  | (n) |
| --- | --- | --- |
| **Gender** |  |  |
|  | Male | 50 |
|  | Female | 100 |
| **Colorectal lesion** |  |  |
|  | CRC | 20 |
|  | SP | 40 |
|  | LGD | 19 |
|  | HGD | 9 |
|  | TA | 35 |
|  | TVA | 16 |

CRC, colorectal cancer; SP, serrated polyp; LGD, low grade dysplasia; HGD, high grade dysplasia; TA, tubular adenoma; TVA, tubulovillous ademona; n, number of patients

**S3 Table.** **Primers for stool-based assays**

| **Gene** | **Sequence 5′ → 3′** | **Reference** | |
| --- | --- | --- | --- |
| *F.nucleatum* (*nusG*) | Forward: CAACCATTACTTTAACTCTACCATGTTCA  Reverse: GTTGACTTTACAGAAGGAGATTATGTAAAAATC | [2] |  |

**S4 Table.** **Primers and probe sets used for quantitative PCR of tissue samples.**

| **Gene** | | **Sequence 5′ → 3′** | **Reference** |
| --- | --- | --- | --- |
| *F. nucleatum (nusG)* | Forward:  CAACCATTACTTTAACTCTACCATGTTCA | | [2] |
|  | Reverse: GTTGACTTTACAGAAGGAGATTATGTAAAAATC | |  |
|  | Probe: TCAGCAACTTGTCCTTCTTGATCTTTAAATGAACC | |  |
| *PGT* | Forward: ATCCCCAAAGCACCTGGTTT | | [2] |
|  | Reverse: AGAGGCCAAGATAGTCCTGGTAA | |  |
|  | Probe: CCATCCATGTCCTCATCTC | |  |

*PGT*, prostaglandin transporter

1. Purcell RV, Pearson J, Aitchison A, Dixon L, Frizelle FA, Keenan JI. Colonization with enterotoxigenic Bacteroides fragilis is associated with early-stage colorectal neoplasia. PloS one. 2017;12(2):e0171602.

2. Flanagan L, Schmid J, Ebert M, Soucek P, Kunicka T, Liska V, et al. Fusobacterium nucleatum associates with stages of colorectal neoplasia development, colorectal cancer and disease outcome. European journal of clinical microbiology & infectious diseases : official publication of the European Society of Clinical Microbiology. 2014;33(8):1381-90.
